# Systematically Deciphering the Pharmacological Mechanism of Fructus Aurantii via Network Pharmacology

**DOI:** 10.1155/2021/6236135

**Published:** 2021-01-21

**Authors:** Qionglong Jin, Jie Lu, Renhui Gao, Jiaying Xu, Xiaoyan Pan, Lichang Wang

**Affiliations:** ^1^Department of Stomatology, Yuyao People's Hospital, Yuyao 315400, Zhejiang, China; ^2^School of Pharmacy, Health Science Center, Xi'an Jiaotong University, Xi'an 710000, Shaanxi, China

## Abstract

Fructus Aurantii (FA) is a traditional herbal medicine that has been widely used for thousands of years in China and possesses a variety of pharmacological effects. However, the active ingredients in FA and the potential mechanisms of its therapeutic effects have not been fully explored. Here, we applied a network pharmacology approach to explore the potential mechanisms of FA. We identified 5 active compounds from FA and a total of 209 potential targets to construct a protein-protein interaction (PPI) network. Prostaglandin G/H synthase 2 (PTGS2), heat shock protein 90 (HSP90), cell division protein kinase 6 (CDK6), caspase 3 (CASP3), apoptosis regulator Bcl-2 (Bcl-2), and matrix metalloproteinase-9 (MMP9) were identified as key targets of FA in the treatment of multiple diseases. Gene ontology (GO) enrichment demonstrated that FA was highly related to transcription initiation from RNA polymerase II promoter, DNA-templated transcription, positive regulation of transcription, regulation of apoptosis process, and regulation of cell proliferation. Various signaling pathways involved in the treatment of FA were identified, including pathways in cancer and pathways specifically related to prostate cancer, colorectal cancer, PI3K-Akt, apoptosis, and non-small-cell lung cancer. TP53, AKT1, caspase 3, MAPK3, PTGS2, and BAX/BCL2 were related key targets in the identified enriched pathways and the PPI network. In addition, our molecular docking results showed that the bioactive compounds in FA can tightly bind to most target proteins. This article reveals via network pharmacology research the possible mechanism(s) by which FA exerts its activities in the treatment of various diseases and lays a foundation for further experiments and the development of a rational clinical application of FA.

## 1. Introduction

Fructus Aurantii (FA, the unripe fruit of *Citrus aurantium* Linn, also known as Zhike) is a traditional herbal medicine widely used for thousands of years in China. Its clinical efficacy has been proven by a plethora of clinical observations and practice [1]. FA exhibits a variety of pharmacological properties, such as antidepressant, antidiabetic, antinephrolithic, and anticarcinogenic properties, enhances gastrointestinal motility, and conveys cardiovascular protective effects [2, 3]. Zhang et al. found that FA is a desirable antidepressant that efficiently reverses depressive-like behaviors [4]. Its usage as a protective herb to treat gastrointestinal dysfunction via accelerated gastric emptying and intestinal transit has also been confirmed by pharmacological studies and clinical trials [5, 6]. It is worth noting that FA exerted a strong effect on symptoms of cardiovascular disease according to recent pharmacology studies. For example, Kang et al. observed that FA exerts distinct anti-ischemic effects via recovery of contractile dysfunction in ischemic hearts [7]. It has been reported that FA reduces portal pressure in portal hypertensive rats by increasing mean arterial pressure and preventing shock via an antifibrinolytic effect [8]. In a recent study, Yang et al. showed that polysaccharides in FA attenuate myocardial ischemia-reperfusion injury by modulating Akt and ERK activity and suppressing oxidative stress [1]. These findings provide evidence for the effects of FA on cardiovascular systems. Other pharmacological effects of FA, such as blood lipid regulation, obesity prevention by modulating gut microbiota, and anti-inflammatory effects, have also been widely reported [9, 10]. However, although many studies have confirmed that FA exhibits various therapeutic effects, the active ingredients and underlying mechanisms of FA have not yet been fully explored, which has complicated the modernization and clinical usage of FA. Thus, it is necessary to identify the bioactive substances of FA and elucidate the mechanisms of action.

Although the widespread use of traditional Chinese medicine (TCM) has increased in the prevention and treatment of diseases, it is difficult to determine the exact pharmacological mechanism with traditional experimental approaches. Herbs contain numerous compounds and complexes and can target many macromolecules, which present a tremendous challenge in conventional pharmacological research. Thus, network pharmacology, an emerging field that integrates computer-aided drug design and bioinformatic analysis, has been rapidly developed to reveal the complex interactions between bioactive ingredients and their related targets [11, 12]. It is a novel and promising method that systematically investigates the interaction networks of compounds, targets, pathways, and diseases to elucidate the potential underlying therapeutic mechanisms of TCM [13]. Several recent studies have indicated that network pharmacology can facilitate the exploration of the effects of TCM at the molecular level from a systematic perspective. Song et al. predicted the potential targets and analyzed the interaction network to identify the potential mechanisms of *Hedyotis diffusa* Willd. for the treatment of prostate cancer [14]. Jiang et al. applied a network pharmacology approach to explore the potential mechanisms of Yiqi Shexue formula and proposed the potential rationale underlying the core herbs and their pharmacological mechanisms in combating primary immune thrombocytopenia [15]. The predicted targets and protective mechanism of JiaWei FoShou San were deciphered by integrating network pharmacology analysis and traditional experimental verification [16]. The antirheumatic mechanism of Qing-Luo-Yin was identified by ingredient preparation, target prediction, enrichment analysis, and network construction [17].

In this study, we applied a network pharmacology approach to explore the comprehensive mechanisms of FA. First, we screened for active compounds of FA by evaluating their oral bioavailability (OB) and drug-likeness (DL). We next identified the targets of the active ingredients in FA via the Traditional Chinese Medicine Systems Pharmacology Database and Analysis Platform (TCMSP) and PharmMapper databases and constructed a compound-target network to analyze the potential interactions between active compounds and potential targets. Gene ontology (GO) and Kyoto Encyclopedia of Genes and Genomes (KEGG) enrichment analyses were performed to investigate the potential mechanism of the active compounds of FA. In addition, compound-target-pathway and compound-target-disease networks were constructed and visualized. Protein-protein interaction (PPI) data were analyzed from the Search Tool for the Retrieval of Interacting Genes (STRING) database to identify the major hubs within the PPI network. Finally, the interaction of compounds and targets was verified using molecular docking. This approach might provide a valuable reference for future pharmacological research and the development of clinical applications of FA.

## 2. Methods

### 2.1. Identification and Screening Strategy of Candidate Components in FA

The candidate ingredients of FA were retrieved from the TCMSP database. Identification of ADME (absorption, distribution, metabolism, and excretion) properties from the TCMSP database was employed to screen the candidate compounds in FA [11, 18, 19]. Currently, DL evaluation (e.g., Lipinski's Rule-of-Five, Opera's rules of DL, and the ROES filter) is integrated into computational drug design/discovery pipelines [20]. In the current study, OB and DL identified the potential bioactive compounds of FA; ingredients with an OB ≥30% and a DL ≥0.18 were selected as for subsequent analysis [21]. The compound structures were saved in mol2 format for further investigation.

### 2.2. Identification of Candidate Targets

To gather information on the interactions between active functional compounds in FA and their associated targets, a multiple targeting strategy that effectively integrates a systematic in silico prediction model and data mining was proposed to identify target proteins of the active compounds. Initially, the targets of bioactive compounds were chosen as the potential targets through the TCMSP servers, which was based on a robust multiple drug-target interaction prediction model. In addition, the optimized structures of active compounds in mol2 format were submitted to PharmMapper to predict the three-dimensional structures of targets in the Protein Data Bank [22]. The lists of predicted targets with a fit score >3.5 were further annotated to screen the putative target list pertaining to activity. All the retrieved targets were converted to their official symbols using the UniProt Knowledge base (UniProtKB) search function (http://www.uniprot.org/) in the protein database.

### 2.3. Network Construction and Analysis

To investigate the pharmacological mechanisms of FA at the system level, compound-target-pathway-disease networks were separately constructed and visualized using Cytoscape 3.6.1 software to comprehensively clarify the complicated relationships among the compounds, targets, diseases, and related pathways [23]. This software allowed for data integration to analyze and visualize complex interaction networks. In these networks, nodes represent compounds, proteins, pathways, and diseases, while the edges indicate their interactions.

### 2.4. Gene Ontology (GO) Analysis

To investigate the meaningful functional annotation and biological properties of the potential targets, GO enrichment analysis was conducted to extract the key GO terms (BP: biological process, MF: molecular function, and CC: cell component) based on the widely used plugin ClueGO in Cytoscape and the platform Enrichr [12]. The target proteins were submitted to Enrichr and ClueGO in text format, and the visualized gene network graphs were analyzed to investigate the target protein functions. The targets that organized and condensed into several functional groups as denoted by their most significant leading term were visualized in the network. The GO terms that had a *p* value of ≤0.05 were regarded as significant and were further pursued.

### 2.5. Kyoto Encyclopedia of Genes and Genomes (KEGG) Pathway Analysis

Enrichr is a web-based platform that includes applications for gene-set libraries, rank-based enriched terms, and various interactive terms. KEGG pathways were enriched and ranked based upon the combined score, which was calculated by the Enrichr platform. An adjusted *p* value threshold of 0.05 was used for pathway discovery. In this study, we chose the top ten KEGG terms and explored their related pathways.

### 2.6. Protein-Protein Interaction (PPI) Networks

The acquired target genes were submitted to STRING (https://STRING-db.org/) to identify the functional protein association networks according to calculation parameters and screening scores [24]. STRING is a widely used platform that contains all publicly available sources of PPI information. The interactions of the overlapping target genes were predicted using the STRING database based on the cutoff criterion of required confidence >0.4. The PPI networks were downloaded and submitted to Cytoscape software to visualize the PPI network.

### 2.7. Molecule Docking

To further evaluate their selectivity, candidate molecules were optimized using SYBYL X2.0 and then docked to predicted target proteins. The crystal structures of these proteins were obtained from the Protein Data Bank (**1**-Akt1: 1h10; **2**-BAX: 2g5b; **3**-BCL2: 2w3l; **4**-caspase3: 1cp3; **5**-IL-2: 1z92; **6**-INSR: 5e1s; **7**-JNU: 1jnm; **8**-MAPK1: 1pme; **9**-MAPK3-ERK: 2zoq; **10**-MAPK8-JNK: 1ukh; **11**-MDM2: 4jrg; **12**-MMP9: 1itv; **13**-NOS2: 3e7g; **14**-PPARA: 1i7g; **15**-PPARG: 6c5q; **16**-PTGS2: 5f19; **17**-RELA: 3qxy; **18**-STAT1: 3wwt; **19**-TGFB1: 3kfd; **20**-tp53: 1aie). Using the biopolymer structure preparation module, the proteins were subjected to the necessary preparation steps before docking was initiated. The Surflex-Dock algorithm follows an empirical scoring system. Based on the total score, Crash, Polar, D-score, G-score, Chem score, and Potential Mean Force (PMF) score, the interaction between the protein and compound (ligand) was studied [25]. The parameters were set to default values. Each ligand was given a rank based on all the scores obtained, and a Total score greater than 3 indicated a good ligand-protein interaction [26, 27].

## 3. Results

### 3.1. Candidate Compounds and Their Putative Target Proteins

**Figure 1:**
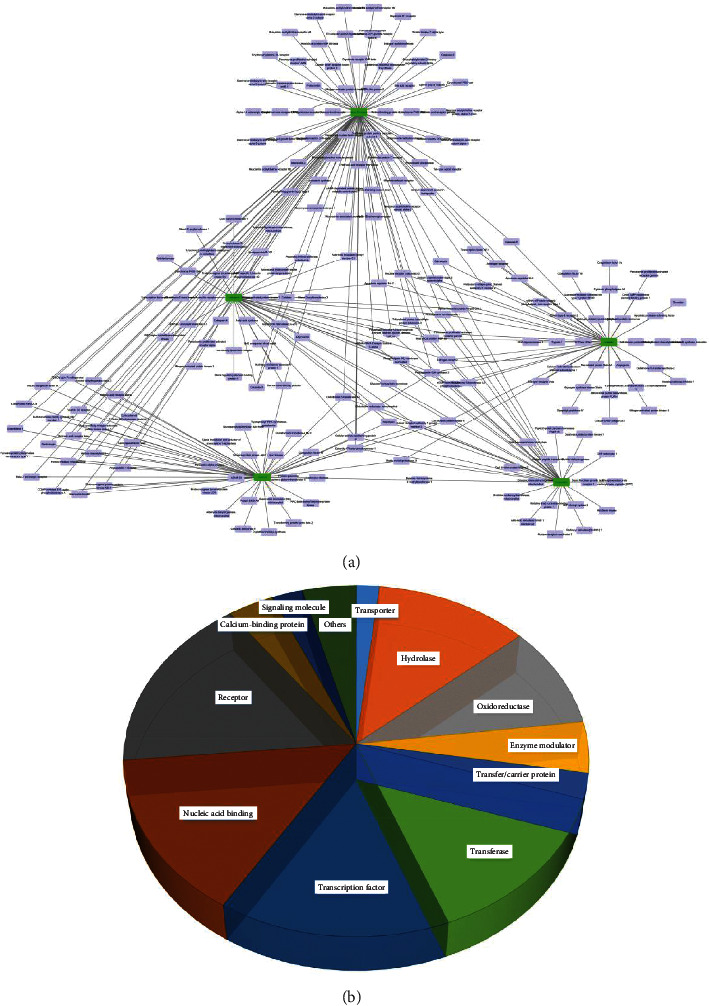
The C-T network of FA and the targets class. (a) The compound in FA and the potential target network. Green nodes represent the candidate compounds; purple represent the predicted targets. (b) The distribution of the candidate targets.

In silico prescreening models were used to identify the main components of FA with favorable pharmacokinetic characteristics; total of 5 candidate compounds of FA were obtained after ADME identification (Table 1). Based on inverse docking and pharmacophore matching platforms, we identified 286 putative target proteins and established a network to elucidate the relationships among the candidate compounds and their putative targets (Figure 1(a)). The detailed information of this network is shown in Table S1. The network showed that the component connected to most targets was beta-Sitosterol (degree = 102), nobiletin (degree = 50), naringenin (degree = 50), and marmin (degree = 50), indicating that these compounds are probably the most critical components in FA. The C-T network contains 214 nodes and 291 ligand–target interactions. The average number of targets per compound is 57.2, in accordance with TCM composition and the features of target interaction.

In addition, we found that many target genes were affected by multiple compounds, implying the complex interplay of herbs. For instance, prostaglandin G/H synthase 2 (PTGS2) was modulated by beta-sitosterol, nobiletin, naringenin, marmin, and hesperetin. Similarly, cellular retinoic acid-binding protein 2 (CHRM2), prostaglandin G/H synthase 1 (PTGS1), heat shock protein 90 (HSP90), cell division protein kinase 6 (CDK6), sex hormone-binding globulin (SHBG), caspase 3 (CASP3), glutathione S-transferase A1 (GSTA1), apoptosis regulator Bcl-2 (Bcl-2), nuclear receptor coactivator 2 (NCOA2), matrix metalloproteinase-9 (MMP9), and estrogen receptor (ER) can also be regulated by more than two ingredients.

The distribution of the biochemical classification indicates that the target space mainly consists of hydrolase, oxidoreductase, enzyme modulator, transferase, transcription factor, nucleic acid binding, receptor, calcium-binding protein, and signaling molecule. Remarkably, the collected targets of FA are enriched in receptor (17.1%), nucleic acid binding (15.2%), transcription factor (15.2%), and transferase (14.6%). Among the targets, 28 targets are receptors, 25 are transcription factor, and 25 are nucleic acid binding (Figure 1(b)).

### 3.2. GO Enrichment Analysis

**Figure 2:**
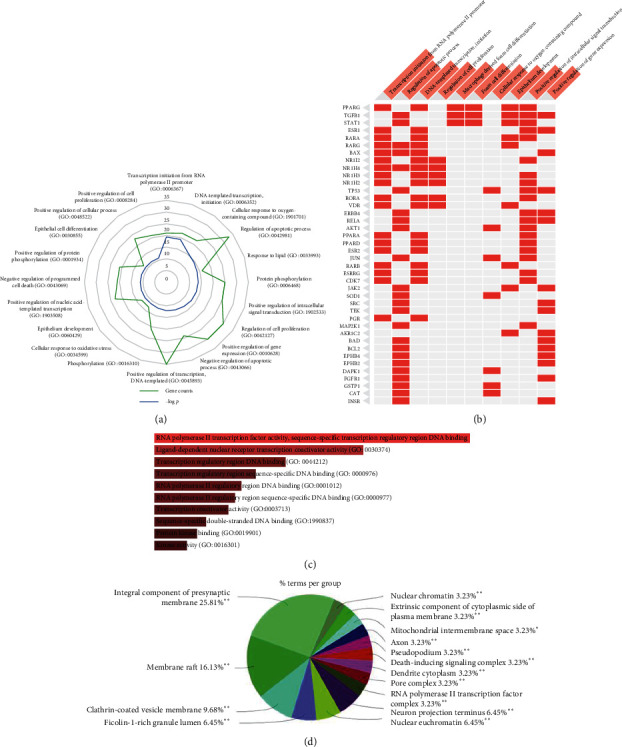
The GO analysis of predicted targets of FA. (a) Enrichr analysis was performed to identify the most significantly enriched BP terms. (b) Enriched BP terms are the columns, input genes are the rows, and cells in the matrix indicate if a gene is associated with a term. (c) The MF analysis of predicted targets of FA. (d) ClueGO was used to identify the most significantly enriched CC terms. The top GO functional categories with false discovery rate (FDR) <0.05 were selected. ^∗^^∗^*P* < 0.01.

To further explore the biological effects of FA as a therapy or cure, we performed GO enrichment analysis of the target proteins. First, we identified and visualized the biological processes of the FA compound targets. As Figure 2(a) shows, the radar map represents the top 20 related biological processes with adjusted *p* values of <0.05. According to their *p* value, the most obvious biological processes were transcription initiation from RNA polymerase II promoter (GO: 0006367) and DNA-templated transcription and initiation (GO: 0006352). According to their gene counts, the most obvious biological processes were positive regulation of transcription DNA-templated (gene counts = 35), regulation of apoptotic process (gene counts = 33), regulation of cell proliferation (gene counts = 30), and positive regulation of gene expression (gene counts = 30). As Figure 2(b) shows, red cells in the matrix represent the 40 potential targets of FA and their related biological processes. The most frequently occurring protein targets were PPARG, TGF-*β*1, STAT1, BAX, and TP53.

As was shown in Figure 2(c), the top five MF enrichment terms (adjusted *p* value <0.05) included (1) RNA polymerase II transcription factor activity and sequence-specific transcription regulatory region DNA binding (GO: 0001133), (2) ligand-dependent nuclear receptor transcription coactivator activity (GO: 0030374), (3) transcription regulatory region DNA binding (GO: 0044212), (4) transcription regulatory region sequence-specific DNA binding (GO: 0000976), and (5) RNA polymerase II regulatory region DNA binding (GO: 0001012). The top 20 significantly enriched MF terms are presented in Table S2.

As was shown in Figure 2(d), we identified and visualized the top enrichment results in the related items of cellular component, which included membrane microdomain, membrane raft, membrane region, plasma membrane raft, axon, postsynaptic membrane, integral component of presynaptic membrane, intrinsic component of presynaptic membrane, presynaptic membrane, integral component of synaptic membrane, and intrinsic component of synaptic membrane. These above observations are valued in improved understanding of the mechanism of FA.

### 3.3. Pathway Enrichment Analysis

**Figure 3:**
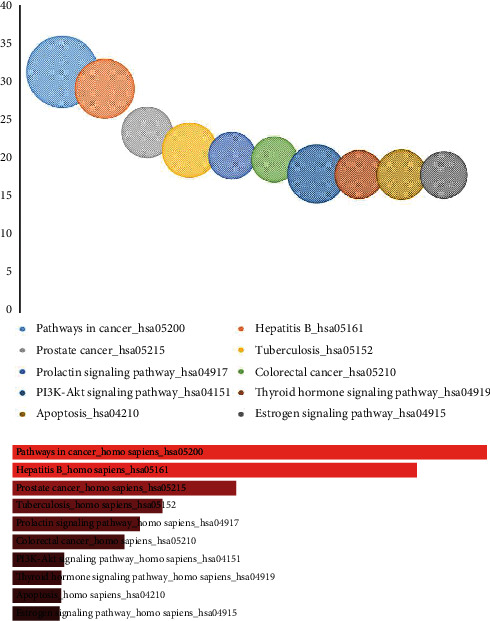
The KEGG enrichment analysis of predicted targets of FA. Pathways that had significant changes of FDR <0.05 were identified. Size of the spot represents number of genes. The enrichment results are also displayed as a bar graph that shows the top 10 enriched terms. The bar graph provides a visual representation of how significant each term is based on the overlap with the predicted targets of FA, and the longer bars and lighter colored bars mean that the term is more significant based on combined score ranking.

To investigate the underlying mechanism of FA, the potential targets of FA were further mapped to pathways. We obtained a total of 123 pathways that belonged to several categories, including human diseases, environmental information, organismal systems, and cellular processes, among others; the top 10 significantly enriched pathways are presented in Figure 3. In cancer-related diseases, several pathways have been verified as important and accurate target pathways, such as pathways in cancer (hsa05200), hepatitis B pathway (hsa05161), prostate cancer (hsa05215), apoptosis (hsa04210), tuberculosis (hsa05152), PI3K-Akt signaling pathway (hsa04151), estrogen signaling pathway (hsa04915), insulin signaling pathway (hsa04910), ErbB signaling pathway (hsa04012), and AGE-RAGE signaling pathway (hsa04933) were enriched. The detailed KEGG information is shown in Table S2.

Among them, pathways in cancer (gene counts = 39, combined score = 152.4335) has the highest combined score, which implies the potential effect of FA in the treatment and prevention of cancer. In addition, specific signaling pathways, including the estrogen, PI3K-Akt, AGE-RAGE, ErbB, Ras, FoxO, and TNF signaling pathways, are also capable of regulating anti-inflammatory, neuroprotective, and antioxidative effects. This KEGG enrichment result showed that FA was highly involved in the regulation of angiogenesis, cell differentiation, migration, apoptosis, invasion, and proliferation.

### 3.4. Compound-Target-Pathway and Compound-Target-Disease Networks Analysis

**Figure 4:**
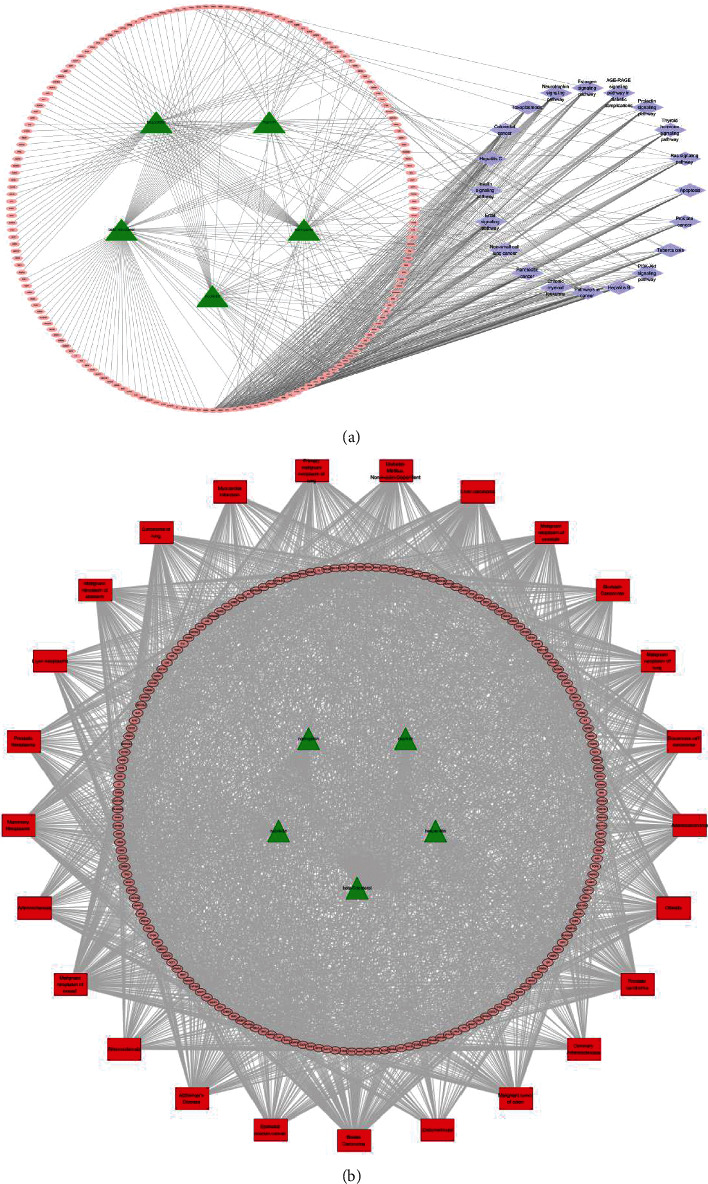
The C-T-P and C-T-D networks. (a) C-T-P network: the compound-potential targets-pathways network and nodes represent compounds, targets, and pathways. Green nodes represent the candidate compounds; pink represents the targets; purple represents the enriched pathways. (b) C-T-D network: a compounds-targets-diseases network and nodes represent compounds, targets, and diseases. Green nodes represent the candidate compounds; pink represents the targets; red represents various diseases.

The compound-target-pathway network, which contains 201 nodes and 615 edges (Figure 4(a)), and the compound-target-disease network, which contains 214 nodes and 2438 edges (Figure 4(b)), were constructed based on the significantly enriched pathways or diseases and targets that regulated these pathways or diseases. The triangles represent bioactive compounds, the circles represent target genes, the rhombuses represent pathways, and the rectangles represent diseases in the network. The top three compound nodes linked to the most target nodes were beta-sitosterol, nobiletin, and naringenin. Interestingly, the network diagram suggested that PTGS2 had the highest maximum betweenness centrality and was the core target gene. Several other genes also had larger betweenness centrality, such as MMP9, PTGS1, CDK6, and BCL2; thus, they might be key target genes for FA against other diseases. Taken together, our results suggested that FA exerts therapeutic effects in a multipathway, multitarget, and overall cooperative manner.

### 3.5. Protein-Protein Interactions (PPI) Network

**Figure 5:**
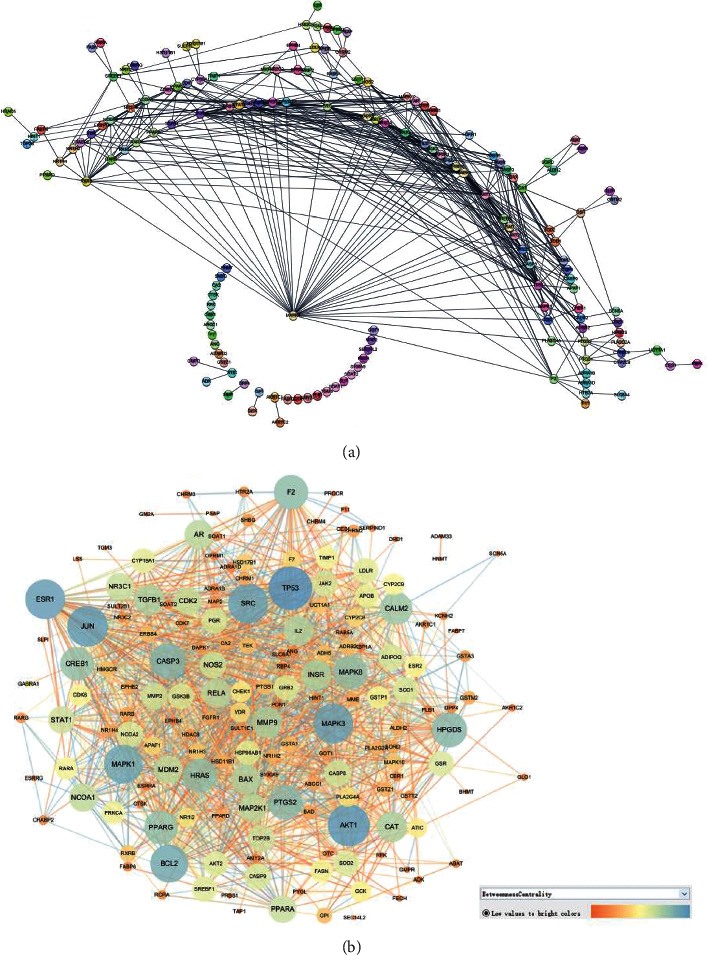
Network pharmacology analysis through the protein-protein interaction of predicted protein targets of FA. (a) The network nodes were predicted proteins and the edges represented the functional associations. (b) The colors of nodes were changed with the betweenness centrality, the size of nodes was changed with the degree, and the colors of edge were changed with the combined score. The degree of each node was defined by the number of connections that the node has. The betweenness centrality of a node reflects the amount of control that this node exerts over the interactions of other nodes in the network.

The PPI network was constructed by mapping the putative targets of FA into the STRING platform (Figure 5(a)). After excluding isolated nodes, the PPI data were imported into Cytoscape, and the layout network was reconstructed for better visualization and understanding (Figure 5(b)). The PPI network consisted of 169 proteins and 1534 edges and was analyzed with the network analyzer plugin. The network diameter was 5, and the average number of neighbors was 18.15. In the PPI network, 22 nodes were selected as major nodes, namely, TP53, SRC, JUN, MAPK1, MAPK3, CALM2, ESR1, AKT1, BCL2, PPARA, TGF beita-1, CDK2, F2, MMP-9, NOS2, CASP-3, CASP-8, STAT1, PPARG, PTGS2, BAX, and HPGDS. The top targets with the greatest degree were TP53 (degree = 75), AKT1 (degree = 71), JUN (degree = 67), ESR (degree = 65), MAPK3 (degree = 65), SRC (degree = 65), MAPK1 (degree = 62), BCL2 (degree = 60), CASP3 (degree = 55), and PTGS2 (degree = 52). Thus, this central target is likely to play a key role in the therapeutic activities of FA.

### 3.6. Molecule Docking

**Figure 6:**
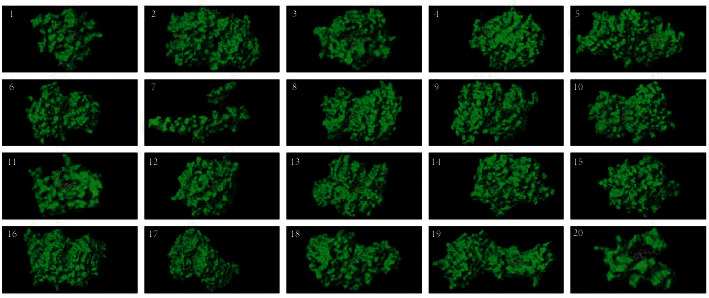
The molecule docking results of 5 bioactive compounds in FA with 20 key target proteins in enriched pathways and PPI network. The list of potential target proteins is as follows: 1-Akt1; 2-BAX; 3-BCL2; 4-caspase 3; 5-IL-2; 6-INSR; 7-JNU; 8-MAPK1; 9-MAPK3-ERK; 10-MAPK8-JNK; 11-MDM2; 12-MMP9; 13-NOS2; 14-PPARA; 15-PPARG; 16-PTGS2; 17-RELA; 18-STAT1; 19-TGFB1; 20-tp53.

A molecular docking study was performed to verify the interaction of bioactive compounds and their potential targets. The docking affinities of the 5 compounds for 20 potential target proteins that were key nodes in the PPI network are presented in Table 2. Detailed interaction information for the active compounds and their related target proteins is shown in Figure 6. It is clear that nobiletin, marmin, hesperetin, beta-sitosterol, and naringenin could bind the most targets via hydrogen bonds, which was consistent with the results obtained from the pharmacophore matching platform PharmMapper.

## 4. Discussion

The noticeable therapeutic effects of FA have been confirmed by multiple clinical and pharmacological studies. However, the mechanism of FA from the perspective of modern medicine has not yet been fully clarified. The network pharmacology method provides a novel approach and systematic perspective for the study of herbs. In the present study, we applied network pharmacology to explore the correlation between effective components of FA and their potential targets and to analyze the compound-target-pathway-disease networks. GO and KEGG enrichment analyses were conducted, PPI networks were constructed, and molecular docking was performed to systematically explore the mechanism of action of FA in the treatment of various diseases.

We employed the ADME evaluation system in TCMSP to obtain 5 active ingredients: nobiletin, marmin, hesperetin, beta-sitosterol, and naringenin. Recent studies have shown that some of the active ingredients in FA have multiple biological activities, which confirms the bioinformatic data produced in our study and highlights the credibility of network pharmacology approach. Nobiletin, a polymethoxy flavonoid, has been proven to exert multiple beneficial activities, including anticarcinogenic, anti-inflammatory, antiasthmatic, and neuroprotective effects [28]. A recent study showed that nobiletin protects against ischemia-reperfusion injury by attenuating oxidative stress and inflammation [29]. Hesperetin, a member of the flavanone class of flavonoids, has been widely studied for its anticancer, antioxidant, and anti-inflammatory properties [30]. Hesperetin exerts anticancer functions by inhibiting cell proliferation, suppressing invasion and inducing apoptosis in different cancer types. In addition, Jo et al. demonstrated that hesperetin blocked neuroinflammation in microglia by regulating the expression of proteins associated with oxidative stress, the inflammatory response, and apoptosis [31]. Naringenin has been proven to have antioxidant, antitumor, antiviral, antibacterial, anti-inflammatory, antiadipogenic, and cardioprotective capacities in in vitro, in vivo, and clinical studies [32, 33].

By searching the direct target proteins from the TCMSP database and the pharmacophore matching platform PharmMapper, 286 target proteins for the 5 identified active compounds in FA were obtained. Through the compound-target network, we found that the compounds exert their therapeutic effects by modulating multiple protein targets, which demonstrated the synergy of TCM strategies featuring multicomponent and multitarget characteristics. For instance, PTGS2 expression and activity were modulated by beta-sitosterol, nobiletin, naringenin, marmin, and hesperetin, indicating its critical role in mitigating disease progression. PTGS2, also known as COX-2, is widely expressed in human tissues and plays a key role in the inflammatory response [34]. The association of PTGS2 with cancer, cardiovascular disease, and multiple diseases has been confirmed in a variety of studies. The inhibition and silencing of PTGS2 could suppress the inflammatory response, thereby eliciting protective effects [35]. HSP90, an evolutionarily conserved molecular chaperone, has been proven to play important roles in diverse fundamental cellular processes, including cell cycle progression, cell proliferation, and other cancer-associated hallmark features [36]. In recent years, HSP90 has emerged as an important target in cancer therapeutics, and some HSP90 inhibitors are currently in preclinical development or phase 1 trials in cancer patients. Inhibitors of CDK6 are also being tested in clinical trials for several types of cancer, with promising results [37]. Caspase 3 and Bcl-2 have been acknowledged as classic targets of regulating apoptosis [38]. MMP9 is an important mediator and marker of inflammation and tissue remodeling. Besides, MMP9 expression correlates with abnormal collagen deposition associated with chronic diseases, including various cancers and cardiovascular diseases [39]. Thus, the bioactive compounds from FA and their interactions with PTGS2, HSP90, CDK6, caspase 3, Bcl-2, and MMP9 may be key in the treatment of cardiovascular disease and various cancers.

According to the results from the GO enrichment analysis, we found that targets of FA are frequently involved with the BP “transcription initiation from RNA polymerase II promoter,” “DNA-templated transcription, initiation,” “positive regulation of transcription, DNA-templated,” “regulation of apoptotic process,” and “regulation of cell proliferation.” These BP terms have been proven to be associated with various cancers. Disruptions in apoptotic processes and their failure to resolve are firmly established as central to the progression of cancers [40]. Our results have shown that the protective effects of FA may be related to the regulation of apoptosis, thereby suppressing cancer. One of the key contributors to the occurrence and development of cancer is cell proliferation. In this study, our results suggested that FA is involved in the BP “regulation of cell proliferation.” Tongue squamous cell carcinoma (TSCC) is a major type of oral cancer that is characterized by remarkably aggressive biological behavior with high incidences of lymph node and distant metastasis [41]. Zhang et al. identified a total of 1050 upregulated and 702 downregulated differentially expressed genes in TSCC from clinical samples and found that the genes were significantly enriched in the biological processes “regulation of transcription from RNA polymerase II promoter,” “negative regulation of cell proliferation,” and “apoptotic process” [42]. Combining their bioinformatic analysis with our virtual screening results, it is possible that FA can exert its therapeutic effects in TSCC by regulating multiple related pathological processes.

Our results suggest that the enriched KEGG pathways of FA targets are closely associated with various pathophysiological conditions, such as cardiovascular disease, diabetes mellitus, inflammation, and various types of cancer. According to the combined score or gene counts, the enriched pathways included pathways in cancer as well as prostate cancer, colorectal cancer, PI3K-Akt, apoptosis, and non-small-cell lung cancer signaling pathways. Numerous studies have shown that pathways in cancer and the PI3K-Akt pathway play important roles in the genesis and growth of TSCC [43]. Various studies have suggested that nobiletin and hesperetin suppress cell viability and inhibit cancer cell scattering and cytoskeletal changes by modulating PI3K/Akt pathway activity [44–46]. Combining established research and the results of the present study, we hypothesized that various compounds in FA may be important contributors to the treatment and prevention of a diverse range of cancers through those biological pathways. Previous studies have provided enormous evidence linking the PI3K-Akt and apoptosis pathways to cardiovascular diseases, including atherosclerosis, hypertension, and myocardial ischemia [47]. The antioxidative, anti-inflammatory, and antiapoptotic effects of the bioactive compounds in FA may lead to a protective effect in cardiovascular tissues by regulating the PI3K-Akt and apoptosis pathways [48]. Based upon the above analyses, we believe that the bioactive components of FA play important roles in the therapeutic effects of FA by regulating the aforementioned signal transduction pathways.

As predicted by the PPI network, the main hubs, such as TP53, AKT1, caspase 3, ERK, PTGS2 BCL2, and BAX, are likely key nodes regulated by FA in the treatment of diseases. Most of these targets have already been verified to be significantly associated with cardiovascular diseases and different cancers. To further validate this position, we performed molecular docking to examine the interactions between the bioactive components of FA and the potential targets in the PPI network. Our docking results showed that the 5 bioactive compounds in FA can tightly bind to most target proteins via hydrogen bonds, which implies that FA exerts its therapeutic effects through those target proteins. In particular, caspase 3 and BAX/BCL2 have been shown to be mutated in multiple cancers and are considered important therapeutic targets. Ma et al. found that nobiletin exerts an inhibitory effect on hepatic cancer cells via modulation of caspase 3 and BAX/BCL2 both in vitro and in vivo, which is consistent with our results [49]. Akt and ERK were found to be regulated by nobiletin in cancer cells [50]. In addition, hesperetin has been shown to modulate the expression of both ERK and Akt in the treatment of cardiovascular diseases and cancers [51, 52]. All these studies together with our network pharmacology results support the conclusion of the network prediction and demonstrated a successful practice of network pharmacology in the identification of the underlying mechanism of FA.

## 5. Conclusion

In conclusion, we explored the mechanisms of action and molecular targets of specific components of FA against cardiovascular diseases and cancers from a systematic perspective using network pharmacology. Nobiletin, marmin, beta-sitosterol, hesperetin, and naringenin were identified and shown to regulate the predicted potential targets associated with different diseases. The bioinformatic analysis was in accordance with current research on FA, especially with regard to the anticancer properties of this herb. In addition, the biological processes and signaling pathways involved in the treatment of FA were identified. TP53, AKT1, caspase 3, MAPK3, PTGS2, and BAX/BCL2 were determined to be related key targets in the enriched pathways and the PPI network. However, more experimental studies are warranted to validate our hypotheses, which will lay the foundation for further experimental research and the development of rational clinical applications of FA.

## Figures and Tables

**Table 1:** The final selected compounds in FA for analysis.

Mol ID	Molecule name	MW	OB (%)	DL	Structures
MOL013381	Marmin	332.43	38.23	0.31	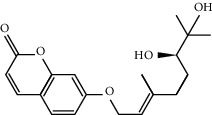

MOL002341	Hesperetin	302.3	70.31	0.27	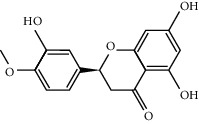

MOL000358	Beta-sitosterol	414.79	36.91	0.75	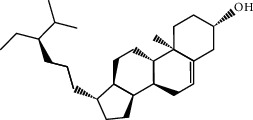

MOL004328	Naringenin	272.27	59.29	0.21	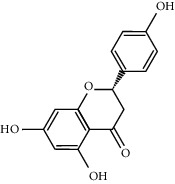

MOL005828	Nobiletin	402.43	61.67	0.52	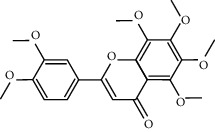

MW: molecular weight; OB: bioavailability; DL: drug-likeness.

**Table 2:** The molecule docking results of 5 bioactive compounds in FA with 20 key target proteins in enriched pathways and PPI network.

No.	Target	Compound	Total_score	Polar	D_SCORE	PMF_SCORE	G_SCORE	CHEMSCORE
1	akt1	Marmin	4.495	3.2292	78.6441	28.6123	−95.0201	−17.5801
1	akt1	Nobiletin	3.4359	0.0001	−96.4127	23.3132	101.7392	−15.7293
1	akt1	Hesperetin	3.5884	2.1784	−70.5591	−5.9415	2.3461	−16.1223
1	akt1	Beta-sitosterol	3.6664	0	−89.2207	24.3479	−181.0379	−17.2961
2	BAX	Beta-sitosterol	5.5461	1.143	−97.1403	−24.5791	−206.5085	−18.2752
2	BAX	Marmin	5.4524	4.0533	−78.1257	−20.6924	−127.0861	−21.5549
2	BAX	Nobiletin	4.5891	1.7811	−109.39	−26.2403	109.932	−19.5921
2	BAX	Naringenin	3.1984	2.4928	−71.2367	−38.9523	−35.6372	−14.6295
2	BAX	Hesperetin	3.5256	5.1405	−74.2037	−59.4876	−69.9864	−17.9244
3	BCL2	Beta-sitosterol	5.3762	0	−96.9549	−17.8952	−209.9829	−25.8636
3	BCL2	Nobiletin	4.7056	1.0474	−96.763	−58.8837	94.8352	−21.0136
3	BCL2	Marmin	4.3354	3.0421	−106.7905	−27.758	−146.1188	−23.0843
3	BCL2	Naringenin	3.3221	0.9947	−81.0599	−53.2372	−81.0883	−23.3243
3	BCL2	Hesperetin	3.9525	2.6188	−73.0159	−28.3856	−36.0666	−19.3607
4	Caspase3	Marmin	5.8252	2.4741	−95.6816	5.4573	−151.9125	−19.8024
4	Caspase3	Beta-sitosterol	4.734	0	−81.6483	7.684	−173.8715	−17.9239
4	Caspase3	Hesperetin	4.2682	4.0141	−71.4363	−17.3613	−25.9661	−21.7364
4	Caspase3	Nobiletin	3.2492	0.0667	−79.6558	−10.0751	100.3961	−16.2548
5	IL-2	Marmin	5.143	2.3782	−112.1538	−20.3672	−143.7453	−23.9844
5	IL-2	Hesperetin	4.7465	3.4248	−104.394	−57.2491	−15.8278	−29.2129
5	IL-2	Beta-sitosterol	4.7142	0	−110.0503	−14.3174	−194.839	−23.3021
5	IL-2	Naringenin	4.1468	4.6059	−70.7242	−55.6216	−80.0299	−26.0309
5	IL-2	Nobiletin	3.3235	0.7416	−113.2679	−36.0754	69.38	−21.4465
6	INSR	Nobiletin	7.32	2.1896	−136.7332	−36.1429	−24.48	−31.6037
6	INSR	Marmin	6.8185	1.3857	−129.3047	−4.3079	−203.1548	−23.0143
6	INSR	Beta-sitosterol	6.2502	0.0001	−113.6603	14.6217	−243.5301	−25.6579
6	INSR	Hesperetin	5.3984	1.7708	−111.7273	−11.1225	−91.8197	−26.4406
6	INSR	Naringenin	5.3693	4.9418	−99.3637	−14.0554	−80.9171	−30.0892
7	JUN	Nobiletin	3.447	0.9679	−94.486	−46.3401	78.5798	−15.2422
8	MAPK1	Hesperetin	6.9655	2.359	−111.0745	−31.8979	−49.5332	−23.3408
8	MAPK1	Beta-sitosterol	6.1293	0	−121.7376	−12.4424	−246.2772	−29.5872
8	MAPK1	Marmin	6.0645	1.024	−124.8175	−17.8817	−166.258	−22.868
8	MAPK1	Nobiletin	6.0374	0.9802	−146.2193	−44.8591	−3.2415	−27.7294
8	MAPK1	Naringenin	5.4443	4.031	−99.4262	−36.7299	−72.162	−30.6483
9	MAPK3-ERK	Hesperetin	6.8921	3.4058	−120.879	−41.0021	−165.2275	−30.177
9	MAPK3-ERK	Nobiletin	6.6601	0.0377	−176.986	−40.9357	−95.3249	−28.1313
9	MAPK3-ERK	Marmin	6.2659	1.7538	−141.8156	−14.1414	−225.3841	−29.4064
9	MAPK3-ERK	Beta-sitosterol	5.1	0	−140.8146	−20.9087	−295.3861	−32.2688
9	MAPK3-ERK	Naringenin	4.6238	2.1294	−103.9795	−29.7774	−128.7848	−25.6133
10	MAPK8-JNK	Nobiletin	6.0558	0.0023	−140.7214	−35.64	−34.6316	−22.6096
10	MAPK8-JNK	Marmin	5.3165	2.5556	−110.4865	−39.4418	−184.4412	−22.0579
10	MAPK8-JNK	Hesperetin	4.8816	2.2786	−108.6111	−15.7012	−32.2504	−17.4629
10	MAPK8-JNK	Beta-Sitosterol	4.5166	0.0331	−96.7772	12.0169	−189.351	−20.465
10	MAPK8-JNK	Naringenin	3.612	2.3254	−81.6187	−32.0155	−14.8548	−19.8751
11	MDM2	Beta-sitosterol	6.0491	0	−98.8408	14.5661	−246.6552	−25.504
11	MDM2	Naringenin	5.1497	1.8488	−90.5226	−18.7392	−34.3502	−27.6954
11	MDM2	Nobiletin	4.9946	1.3229	−106.0889	−36.2829	74.8539	−23.319
11	MDM2	Hesperetin	4.8191	3.0306	−81.2096	−3.1948	26.1109	−21.3567
11	MDM2	Marmin	4.1553	2.1009	−75.3468	−27.7195	−132.9105	−19.2361
12	MMP9	Nobiletin	5.6958	1.4643	−131.7484	−51.7425	8.4283	−24.3482
12	MMP9	Naringenin	5.6928	2.3751	−128.1282	2.7271	−104.5691	−25.6595
12	MMP9	Marmin	5.6757	5.0882	−105.634	−27.9194	−141.3094	−31.1223
12	MMP9	Hesperetin	5.1947	3.0769	−122.0312	−31.7337	−25.5357	−28.2245
12	MMP9	Beta-sitosterol	3.0945	0	−120.8962	11.4655	−258.8751	−23.2881
13	NOS2	Marmin	7.5303	3.6446	−129.6716	−48.9364	−205.402	−27.5053
13	NOS2	Beta-sitosterol	6.3128	0	−120.5563	−20.8855	−228.3523	−23.3981
13	NOS2	Nobiletin	5.6196	1.1805	−120.3695	−62.8354	61.0866	−21.6513
13	NOS2	Naringenin	4.5252	4.5571	−72.3596	−64.6221	−97.9561	−19.6906
13	NOS2	Hesperetin	3.7235	1.0814	−109.4896	−40.1257	34.373	−16.886
14	PPARA	Nobiletin	7.4294	1.8613	−157.0217	−41.9069	−58.5104	−40.4642
14	PPARA	Marmin	7.19	2.2106	−143.5558	1.9991	−231.1587	−32.7461
14	PPARA	Beta-sitosterol	6.5938	0.0003	−168.643	−7.5792	−352.8443	−42.7365
14	PPARA	Naringenin	4.8729	1.8127	−99.2233	−13.3581	−136.9647	−27.7053
14	PPARA	Hesperetin	4.7234	3.3476	−102.3979	−9.1738	−58.2356	−27.821
15	PPARG	Beta-sitosterol	7.3003	0	−151.8674	−13.6812	−305.0754	−38.1467
15	PPARG	Nobiletin	7.2679	0.8344	−140.1593	−36.7598	−34.6353	−27.4784
15	PPARG	Marmin	6.9022	2.5964	−120.8649	−21.4162	−179.4292	−26.9327
15	PPARG	Naringenin	5.0198	2.3445	−87.7577	−31.5021	−55.8475	−25.7127
15	PPARG	Hesperetin	4.8252	1.1863	−101.4288	−9.1484	−38.0543	−21.1472
16	PTGS2	Nobiletin	7.2479	1.0839	−163.8655	−120.8833	−16.5227	−33.2436
16	PTGS2	Marmin	6.9933	3.7462	−157.0198	−70.7414	−228.8652	−34.3804
16	PTGS2	Hesperetin	6.4127	2.2755	−135.8122	−72.2395	−75.5947	−29.1226
16	PTGS2	Beta-sitosterol	4.9704	0	−118.7718	−69.402	−224.0666	−24.5951
16	PTGS2	Naringenin	4.6218	2.1139	−96.6225	−79.4178	−64.0067	−23.0482
17	RELA	Nobiletin	7.3942	0.0007	−159.6319	−73.3286	12.1047	−31.8051
17	RELA	Beta-sitosterol	6.9688	0.0023	−144.1068	−21.2933	−267.9913	−33.3955
17	RELA	Marmin	6.8068	1.1518	−149.5363	−56.5648	−187.9849	−32.1388
17	RELA	Naringenin	6.2223	3.7205	−97.839	−29.3891	−94.2409	−28.2748
17	RELA	Hesperetin	6.2113	3.1391	−113.119	−55.0852	−37.7086	−30.6853
18	STAT1	Marmin	5.9184	3.2531	−91.332	−13.5088	−104.9859	−19.2249
18	STAT1	Hesperetin	5.0082	4.0781	−69.5886	−30.7653	20.4565	−19.866
18	STAT1	Nobiletin	4.6844	1.1218	−108.4174	−10.5814	66.444	−20.083
18	STAT1	Beta-sitosterol	4.6119	0.0009	−105.8922	26.0073	−233.4198	−21.5861
19	TGF-b1	Marmin	5.8231	2.8957	−99.5404	−22.0261	−124.0233	−27.7028
19	TGF-b1	Beta-sitosterol	5.6052	0.899	−105.4581	−9.2255	−212.4984	−30.4989
19	TGF-b1	Naringenin	3.1238	2.5232	−61.9906	−6.4646	−6.6706	−21.1536
19	TGF-b1	Nobiletin	3.1565	0	−114.5095	−16.4065	62.8721	−24.9896
19	TGF-b1	Hesperetin	3.7684	3.2655	−63.5655	−25.2219	56.7478	−23.391
20	TP53	Nobiletin	4.8546	1.0887	−102.858	−30.4447	37.7055	−20.3388
20	TP53	Marmin	4.8005	2.8184	−87.9826	−9.346	−149.2093	−21.7584
20	TP53	Beta-sitosterol	4.3815	0	−101.9354	−6.7784	−228.7715	−25.434
20	TP53	Hesperetin	4.2653	2.1424	−68.8398	−10.7935	25.4891	−19.1776
20	TP53	Naringenin	3.7952	2.7744	−50.7836	−24.9575	−6.1211	−14.5913

## Data Availability

We have presented all our main data in the form of figures and additional file. The datasets supporting the conclusions of this article are included within the article.

## Abbreviations

FA: Fructus Aurantii
TCM: Traditional Chinese medicine
TCMSP: Traditional Chinese Medicine Systems Pharmacology
ADME: Absorption, Distribution, Metabolism and Excretion
OB: Predict oral bioavailability
DL: Predict drug-likeness
GO: Gene ontology
PPI: Protein-protein interaction
KEGG: Kyoto Encyclopedia of Genes and Genomes
BP: Biological process
MF: Molecular function
CC: Cell component
TSCC: Tongue squamous cell carcinoma
FDR: False discovery rate.

## Supplementary Materials

Supplementary Materials Table S1: the candidate compounds and putative target proteins of FA. Table S2: the MF and KEGG enrichment analysis of predicted targets of FA.
